# Commentary: Clinical efficacy of plasmid encoding p62/SQSTM1 (Elenagen) in combination with gemcitabine in patients with platinum-resistant ovarian cancer: a randomized controlled trial

**DOI:** 10.3389/fonc.2025.1727892

**Published:** 2025-12-05

**Authors:** Shuyan Li, Guanze Li

**Affiliations:** 1 The Second School of Clinical Medicine, Zhejiang Chinese Medical University, Hangzhou, Zhejiang, China; 2 Zhejiang Chinese Medical University, Hangzhou, Zhejiang, China

**Keywords:** ovarian cancer, ELENAGEN, commentary, randomized controlled trial, BRCA

## 1 Introduction

While Krasny et al. (1) offer important insights into the use of DNA vaccines for treating platinum-resistant ovarian cancer, several methodological limitations must be carefully considered to inform the further development of DNA vaccine-based interventions. The results of this randomized controlled trial (RCT) are clinically encouraging; however, the study’s implementation revealed conceptual and technical limitations that warrant scholarly discussion to guide the design of more robust future trials.

## 2 Critical methodological challenges

### 2.1 Sample size and geographic representation

With a sample size of merely 40 patients and data collected exclusively from a single country, this study’s results should be interpreted with caution. The limited scale and geographic restriction may constrain the generalizability of the findings, making it difficult to extrapolate the outcomes to more diverse populations with varying demographic and genetic backgrounds.

### 2.2 Follow-up duration

Given a median follow-up of only 13.8 months, the current study may not fully capture long-term outcomes, particularly regarding the durability of treatment responses and the occurrence of serious or late-onset adverse events. Future studies with extended follow-up are likely to provide more reliable and clinically meaningful data.

## 3 Novel perspectives for future research

### 3.1 Open-label design considerations

The open-label nature of this trial, though ethically necessary, may introduce both conscious and unconscious biases in outcome assessment, thereby increasing variability in the reported results. Future studies could address these limitations by employing independent blinded reviews or other methodological safeguards to ensure a more objective and reliable endpoint evaluation.

### 3.2 Additional clinical endpoints and biomarker

In order to provide a more comprehensive evaluation of therapeutic benefit, future studies could consider incorporating additional clinically relevant endpoints, including overall survival (OS), in addition to progression-free survival (PFS) and objective response rate. This approach aligns with the design of numerous related RCT (2), many of which have adopted OS as a key endpoint to better assess long-term outcomes.

Moreover, the article suggests that ELENAGEN not only enhances antitumor immune activation but also reduces chronic inflammation, thereby improving the efficacy of chemotherapy. Therefore, collecting data on inflammatory markers such as interleukin-6 (IL-6) and tumor necrosis factor-alpha (TNF-α) in future studies will be essential to elucidate inflammation-related mechanisms.

### 3.3 *BRCA* mutation data collection

We recommend collecting detailed data on *BRCA* mutation types among patients. Analyzing outcomes stratified by *BRCA* status could reveal differences in treatment response and inform more personalized therapeutic strategies. Notably, recent RCT (3) have shown that various *BRCA* mutation subtypes may respond differently to specific treatment modalities, emphasizing the clinical relevance of mutation-specific data. This study suggests that continuous dosing may confer a potential progression-free survival benefit in patients with *gBRCA* wild-type or unknown status.

A previous study (4) has demonstrated that *BRCA2* mutations are significantly associated with poorer OS, highlighting the need for this investigation.

### 3.4 Insufficient animal evidence for ovarian cancer

In previous studies (5), the p26 vaccine demonstrated antitumor efficacy and an absence of toxicity in animal models of Lewis lung carcinoma, B16 melanoma, S37 sarcoma, and Ca755 breast cancer. However, no preclinical experiments have been conducted using ovarian cancer models. Although multiple genomic studies from different countries have demonstrated that ovarian and breast cancers share similarities in genetic mutations and molecular signaling pathways (6, 7), important differences remain in their clinical management (8, 9).

## 4 Discussion

In conclusion, this study provides valuable preliminary evidence on the efficacy of DNA vaccines in platinum-resistant ovarian cancer. Future trials could expand patient cohorts across multiple regions to enhance generalizability and include longer follow-up to better assess long-term outcomes. Incorporating additional endpoints, such as overall survival and objective response rate, would provide a more comprehensive evaluation of therapeutic benefit. Collecting detailed *BRCA* mutation data may help identify subgroups most likely to benefit and guide personalized strategies. Additionally, implementing independent blinded assessments could improve reliability and reduce potential bias. These approaches will collectively help optimize the design and impact of DNA vaccine-based interventions in ovarian cancer. Given that the current clinical trial has been completed, several of these assessments, including biomarker evaluation, should be considered for inclusion in subsequent follow-up studies to further clarify the drug’s biological effects. Thank you for considering these observations, and I look forward to further developments in this promising field.
